# The Heparan Sulfate Mimetic PG545 Modulates T Cell Responses and Prevents Delayed-Type Hypersensitivity

**DOI:** 10.3389/fimmu.2020.00132

**Published:** 2020-02-06

**Authors:** Ievgen O. Koliesnik, Hedwich F. Kuipers, Carlos O. Medina, Svenja Zihsler, Dan Liu, Jonas D. Van Belleghem, Paul L. Bollyky

**Affiliations:** ^1^Division of Infectious Diseases and Geographic Medicine, Department of Medicine, Beckman Center, Stanford University School of Medicine, Stanford, CA, United States; ^2^Department of Clinical Neurosciences, University of Calgary, Calgary, AB, Canada

**Keywords:** heparan sulfate mimetic, PG545, Th17 cells, regulatory T cells, inflammation

## Abstract

The heparan sulfate mimetic PG545 (pixatimod) is under evaluation as an inhibitor of angiogenesis and metastasis including in human clinical trials. We have examined the effects of PG545 on lymphocyte phenotypes and function. We report that PG545 treatment suppresses effector T cell activation and polarizes T cells away from Th17 and Th1 and toward Foxp3+ regulatory T cell subsets *in vitro* and *in vivo*. Mechanistically, PG545 inhibits Erk1/2 signaling, a pathway known to affect both T cell activation and subset polarization. Interestingly, these effects are also observed in heparanase-deficient T cells, indicating that PG545 has effects that are independent of its role in heparanase inhibition. Consistent with these findings, administration of PG545 in a Th1/Th17-dependent mouse model of a delayed-type hypersensitivity led to reduced footpad inflammation, reduced Th17 memory cells, and an increase in FoxP3+ Treg proliferation. PG545 also promoted Foxp3+ Treg induction by human T cells. Finally, we examined the effects of other heparan sulfate mimetics PI-88 and PG562 on lymphocyte polarization and found that these likewise induced Foxp3+ Treg *in vitro* but did not reduce Th17 numbers or improve delayed-type hypersensitivity in this model. Together, these data indicate that PG545 is a potent inhibitor of Th1/Th17 effector functions and inducer of FoxP3+ Treg. These findings may inform the adaptation of PG545 for clinical applications including in inflammatory pathologies associated with type IV hypersensitivity responses.

## Introduction

Heparan sulfate mimetics have emerged as potential therapies against various types of cancer (1–4). These agents are thought to work via inhibition of heparanase (5–7), an enzyme associated with tumor cell invasion and angiogenesis (6, 8, 9). Heparanase is also known to be involved in normal immune cell functions (10–13). In particular, heparanase overexpression facilitates T cell effector functions and migration, as well as macrophage and NK cell activation (12, 14).

The first such inhibitor, PI-88, demonstrated promising results in mouse cancer models (15). However, a phase III clinical trial using PI-88 as an adjuvant in hepatocellular carcinoma therapy did not reach its primary end point (16).

PG545 is a member of a novel class of synthetic heparanase inhibitors, the PG500 family (17, 18). A fully sulfated oligosaccharide attached to a lipophilic moiety, PG545 has improved pharmacokinetics and much weaker anticoagulant activity relative to other heparanase inhibitors, including PI-88 (19). PG545 is reported to function by blocking the catalytic center of heparinase and competing for the HS-binding domain (11, 19). PG545 is currently under evaluation in human clinical trials for use in treating solid tumors after having demonstrated efficacy against tumor angiogenesis and metastasis in multiple animal studies (1, 3, 20, 21). PG545 has also demonstrated efficacy against lymphoma and may also have utility in other, non-neoplastic inflammatory disorders, such as acute kidney injury, atherosclerosis, and viral infection (2, 9, 22, 23). However, it remains unclear how PG545 impacts immune cells, particularly T-lymphocytes. This knowledge is likely to be important, given the key role of T-lymphocytes in cancer immunology and inflammation.

We sought to evaluate the impact of PG545 on T cells and T cell subsets, including pro-inflammatory Th1 and Th17 cells and anti-inflammatory FoxP3+ regulatory T cells (24, 25). To this end, we studied this agent in the context of *in vitro* T cell activation and proliferation, antigenic responses *in vivo*, and in a skin hypersensitivity model.

We report that *in vitro*, PG545 promotes expansion of Treg while suppressing effector T cells, particular Th17 T cells. *In vivo*, PG545 suppresses antigen-specific T cell generation and cytokine production, leading to improvement in a Th17-dependent delayed hypersensitivity model. Together, these data indicate that PG545 has profound effects on T cell polarization and function.

## Materials and Methods

### Mice

C75BL/6J mice for the experiments were obtained from the Jackson laboratories at the age of 8–10 weeks and kept in specific pathogen free facility on a standard chow. HPSE^−/−^ mice were a kind gift of I. Vlodavsky (26). OT-II B6 transgenic mice were from the Jackson Laboratory. Foxp3^gfp^ reporter mice (27) were a kind gift of Dr. Alexander Rudensky. All mice were maintained in a specific pathogen-free AAALAC-accredited animal facility at Stanford University. All experiments were approved by the Stanford IACUC.

### Human Lymphocyte Isolation

Human peripheral blood mononuclear cells (PBMCs) were obtained and isolated as previously described (28). Human naïve CD4 cells were isolated from frozen PBMC using EasySep^TM^ human CD4 isolation kit (Stemcell technologies, Vancouver, Canada). The cells were then resuspended in RPMI 1640 (Invitrogen) supplemented with 10% FBS (Hyclone, Logan, UT), 100 μg/ml Penicillin, 100 U/ml Streptomycin, 50 μM βme, 2 mM glutamine, and 1 mM sodium pyruvate (Invitrogen).

### Mouse Lymphocyte Isolation and Culture

Primary T cells were isolated from spleen and lymph node cell suspensions as previously described (29) using a CD4 isolation kit (Stemcell technologies). For T cell activation studies, 1 × 10^5^ cells were cultured and activated as previously described (30) using plate bound anti-CD3 (145-2C11, Biolegend) and soluble anti-CD28 (37.51, Biolegend) at concentrations of 2 and 1 μg/ml, respectively. Bone marrow derived dendritic cells were differentiated as previously described (28) Briefly, 10 ×10^6^ bone marrow cells were cultured in complete T cell medium (RMPI 1640, 10% FBS, 100 U/ml Streptomycin, 50 μM βme, 2 mM glutamine, and 1 mM sodium pyruvate) for 7 days in the presence of 10 ng/ml mouse GM-CSF and 2 ng/ml IL-4 (Peprotech). On day 3 and 5 fresh medium with cytokines was added. For co-culture experiments 2 ×10^4^ BMDCs were incubated with 1 μg/ml anti-CD3 antibody (145-2C11, Biolegend) and 1 ×10^5^ naïve CD4 T cells.

### Flow Cytometry

Flow cytometry was performed as previously described (31). Antibodies used included anti-CD4-BV785 (RM4-5), anti-CD8-BV711 (53-6.7), anti-CD62L-BV421 (MEl-14), anti-CD44-APC-Cy7 (IM7), anti-CD25-PE-Cy7-(PC61), anti-CD3-PE-Cy7 (17A2), anti-CD69-APC (H1. 2F3), anti-CD154-PE (MR1), all from BioLegend. For Foxp3, RORγt, and T-bet staining, cells were fixed, permeabilized and stained using Foxp3 Transcription factor staining kit (Thermo Fisher Scientific). The antibodies used for nuclear staining were anti-Foxp3-EF660 (FJK-16s; Thermo Fisher Scientific), anti-Ki-67- PECy7 (SolA 15; Thermo Fisher Scientific), anti-T-bet-Alexa Fluor 647 (4B10, BioLegend), anti-RORγt (Q31-378, BD Biosciences). For cytokine staining 5 ×10^6^ cells were stimulated with 50 ng/ml PMA and 500 ng ionomycin (Sigma-Aldrich) in the presence of Brefeldin A (Bioelegend) for 5 h. Cells were fixed using IC buffer (Thermo Fisher Scientific), permeabilized with 0.5% saponin (Sigma-Aldrich) and stained with anti-IFNg-APC (XMG1.12, anti-IL-17-PE (TC11-18H10.1), anti-TNFa-PE (MP6-XT22). For phosphoflow experiments mouse total CD4 T cells were activated for 3 days with or without PG545, rested for 6 h and restimulated with Dynabeads mouse T cell activator (Thermo Fisher Scientific) for 12 h with or with or without PG545. Cells were fixed in Phosphoflow fixation buffer (BD Biosciences), permeabilized with 90% methanol for 30 min on ice and stained with anti-phospho-Erk44/42-APC (Cell Signaling Technology). Flow cytometry was performed using LSRII (BD Biosciences) or Cytek Aurora (Cytek Biosciences) and the sorting was done at the FACS Aria III (BD Biosciences). Data was analyzed using Flowjo 10 software (Treestar Inc.).

### *In vitro* T-Cell Differentiation

Th1 and Th17 cells were induced as previously described (31). In brief, 1 ×10^5^ naïve CD4 cells were activated with anti-CD3 (145-2C11, Biolegend) and anti-CD28 (37.51, Biolegend) antibodies in presence of 50 ng/ml mouse recombinant IL-12 or 5 ng/ml of human recombinant TGFβ (Biolegend) and 25 ng/ml mouse recombinant IL-6 (Peprotech) for 3 days. For iTreg induction cells were cultured with 50 ng/ml of human recombinant TGFβ with 100 IU/ml recombinant IL-2 (Chiron), as previously described (32). For T cell proliferation cells were stained with 5 uM Cell Trace Violet (Thermo Fisher Scientific) in PBS for 15 min at RT, washed with cell culture media, counted and plated as mentioned earlier. Cells were cultured in RPMI 1640 media containing 2.05 mM L-Glutamine, 10% FBS and 1 × penicillin/streptomycin (GE Healthcare). For some experiments MAPK Kinase Inhibitor PD98059 (Sigma Aldrich) was used.

### *In vitro* Suppression Assays

Suppression assays were performed as previously described (33). In brief, iTregs induced either in the absence or presence of PG545 were co-cultured with Cell Trace labeled responder CD4 cells and T cell-depleted splenocytes as antigen presenting cells. Activation was provided by 1 μg/ml of soluble anti-CD3 (145-2C11, Biolegend).

### Ovalbumin Immunization

Naïve CD4 T cells from OT-II mice were stained with eFlour 450 cell proliferation dye (Thermo Fisher Scientific) and 1 ×10^6^ of the cells adoptively transferred via intravenous tail injections into B6 recipients. The next day mice were immunized with 50 μg of OVA protein emulsified in 100 μL alum (Thermo Fisher Scientific).

### Western Blotting

Protein lysate from CTLL2 cells (ATCC) cells was prepared using RIPA buffer (Thermo Fisher Scientific) and 20 μg of protein per sample were separated on a NuPAGE 4–12% Bis-Tris Protein gel (Thermo Fisher Scientific) and blotted onto a 0.22 μm Odyssey nitrocellulose membrane (LI-COR). Phospho Erk1/2 was detected using a pErk antibody Thr202/204 #9101 (Cell Signaling Technology).

### *In vivo* Delayed Type Hypersensitivity Experiments

These experiments were performed as previously described (34). In brief, 8–10 week old mice were sensitized subcutaneously with 200 μg of mBSA (Sigma-Aldrich) emulsified in Complete Freund's Adjuvant (Santa Cruz Biotechnology) and challenged with 200 μg of mBSA solution in the foot pad of a hind limb. Control foot pad was injected with an equal volume of PBS. Footpad swelling was measured starting at day 0 using a digital caliper (Traceable). The reading of the “PBS” foot were subtracted from the “mBSA” foot for each individual mouse. PG545 dissolved in PBS was administered to mice intraperitoneally at a concentration of 400 μg/mouse at the indicated time point.

### Generation of Murine Bone Marrow–Derived Dendritic Cells

BMDC were generated as described (28). Bone marrow from femurs of 6–10 week old mice was flushed out using a 27 G Precision Glide needle (BD Biosciences, Cat. No. 305109). Cells were plated at 1 ×10^6^ cells in 10 ml of media per Petri Dish (Fisherbrand, Cat. No. FB0875711), supplemented with 10 ng/ml of recombinant mouse granulocyte-macrophage colony-stimulating factor (GM-CSF) (STEMCELL Technologies, Cat. No. 78206.1) and 2 ng/ml of recombinant mouse IL-4 (Invitrogen, Cat. No. 14-8041-62). An equivalent amount of fresh media containing cytokines was added 3 days after plating, and 50% of media was changed on day 5 after plating. BMDCs were harvested for use on day 6.

### Induction of EAE

EAE was induced as described previously (35). In brief, C57BL/6J mice were immunized with 200 μg of myelin oligodendrocyte glycoprotein (35–55) (MOG_35−55_) in complete Freund's adjuvant containing 400 ng of mycobacterium tuberculosis H37RA (Difco Laboratories, Detroit, MI). All mice were administered 400 ng of pertussis toxin (List Biological, Campbell, CA) intraperitoneally (i.p.) at 0 and 48 h post-immunization. Mice were monitored daily for clinical symptoms and scored as follows: 0, no clinical disease; 1, tail weakness; 2, hindlimb weakness; 3, complete hindlimb paralysis; 4, hindlimb paralysis and some forelimb weakness; 5, moribund or dead.

### Statistical Analysis

Statistical analysis was performed using Prism software, version 7.0 (GraphPad). Data are presented as mean SD. In samples with Gaussian distribution, an unpaired *t*-test was used to determine significant differences between groups or a 2-way ANOVA was used to identify effects of multiple parameters. A *p* < 0.05 was considered statistically significant.

## Results

### PG545 Inhibits Effector T Cell Activation *in vitro* and *in vivo*

**Figure 1:**
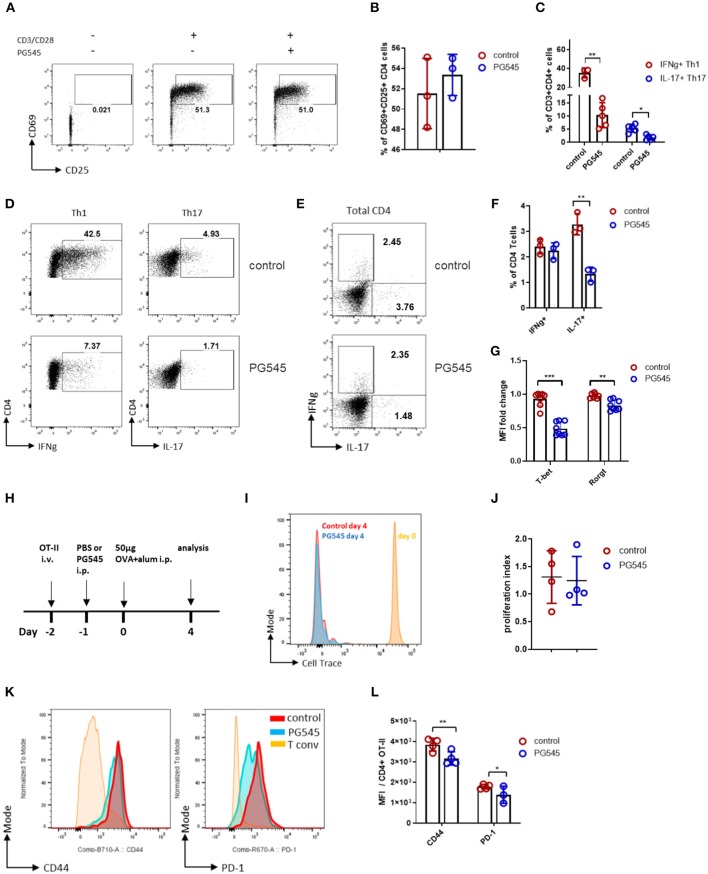
PG545 inhibits effector T cell differentiation. **(A)** FACS plot showing the expression of CD25 and CD69 by mouse CD4 cells in response to CD3/CD28 stimulation in the absence or presence of PG545. **(B)** Cumulative bar diagram of CD25 and CD69 expression. Data are representative of three experiments. **(C)** Aggregate data on T helper cell differentiation in the presence of PG545. Data are representative of three experiments. **(D)** FACS plot of Th1 and Th17 differentiation of naïve T cells in the absence or presence of PG545. **(E,F)** IFNg and IL-17 profile of memory CD4 T cells cultured with PG545 for 48 h. Data are representative of two experiments. **(G)** T-bet and RORgt expression in T cells from Th1 and Th17 polarization, respectively. **(H)** Schematic of the experimental protocol used to generate the data in this figure. **(I)** Overlaid histograms showing the proliferation of transferred OT-II cells in B6 recipients following OVA-alum immunization and injection with either PBS (control) or PG545 (20 μg/g of weight). **(J)** Proliferation index of transferred OT-II cells. **(K)** Representative histograms showing CD44 and PD-1 expression on transferred OT-II cells. **(L)** Aggregated mean fluorescence index (MFI) data from **(K)**. Each data point represents an individual mouse. Data represent mean ± SD; *p < 0.05, **p < 0.01, ***p < 0.001 vs. respective control by unpaired *t*-test or one way ANOVA with multiple comparisons where appropriate.

We first asked whether PG545 affects T cell activation *in vitro*. To test this, we stimulated CD4+ T cells in a polyclonal manner. We observed that PG545 did not inhibit the activation of CD4+ T cells, as measured by the expression of CD69 and CD25 (Figures 1A,B).

We next examined cytokine production by these cells via intracellular staining. We observed that PG545 treatment resulted in altered cytokine production, including reduced IFNg and IL-17 in Th1 and Th17 polarizing conditions, respectively. This is seen for a representative staining example as well as across multiple samples (Figures 1C,D). This was also accompanied by decreased expression of T-bet and RORgt in the setting of PG545 treatment (Figure 1G; Supplemental Figure 1A). PG545 also inhibited IL-17 production by activated total CD4 T cells, while sparing IFNg-competent T cells (Figures 1E,F), suggesting that PG545 acts early on during Th1 differentiation but not on mature Th1 memory cells.

Together, these data indicate that PG545 potently polarizes T helper differentiation away from Th1 and Th17 cells.

We next examined the impact of PG545 on adaptive immunity *in vivo*. To this end, we transferred Cell Trace-labeled, ovalbumin (OVA)-specific OT-II naïve CD4 cells from CD45.1 mice into naïve CD45.2 autologous recipient mice on day−2. These recipient animals then received either a single dose of PG545 or the same volume of phosphate buffered saline (PBS) as a control via intraperitoneal (i.p.) injection on day−1 followed by i.p. immunization with OVA on day 0. Then, on day 4 we euthanized the mice, isolated lymphatic tissue, and examined the proliferation and phenotype of these transferred cells via flow cytometry. A schematic of this protocol is shown in Figure 1H.

Following OVA immunization, transferred OT-II cells underwent rapid proliferation as measured by Cell Trace dilution. Mice that received either PG545 or control injections had equivalent proliferation of OT-II cells (Figures 1I,J). However, these cells from mice that received PG545 had lower expression of CD44 and PD-1 than control OT-II cells. This was seen for a representative staining example (Figure 1K) as well as across samples from multiple mice (Figure 1L). Neither CD25 nor CD69 levels were altered (not shown). This is consistent with the effects of PG545 *in vitro* where we likewise did not observe effects on early T cell activation (Figure 1A). Together, these data suggest that PG545 modestly impacts T cell activation but not proliferation.

We also examined PG545 effects on other aspects of murine health in this system. Mice treated with PG545 developed splenomegaly (Supplemental Figure 1B) a mild albeit not significant weight loss (Supplemental Figure 1C) without an increase in the lymphocyte count (Supplemental Figure 1D).

Together these data demonstrate that PG545 inhibits effector T cell phenotype without affecting their proliferation.

### PG545 Enhances Induction of FoxP3+ Treg *in vitro*

**Figure 2:**
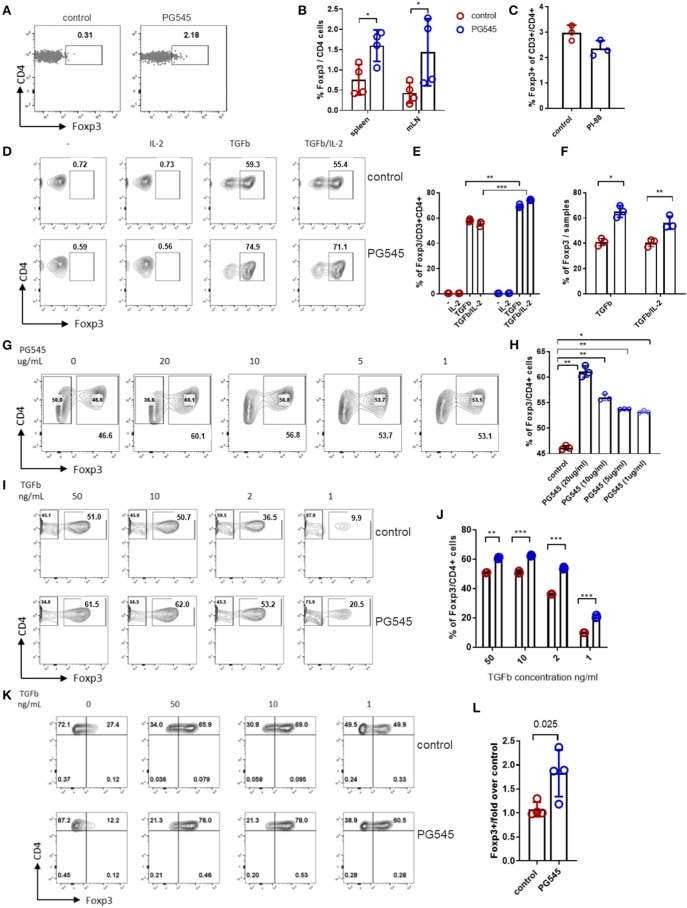
PG545 promotes Treg induction. Treg induction protocols were performed using established protocols and naïve mouse CD4+ cells isolated from spleen and LN of 3–4 month old Foxp3^GFP^ mouse. **(A)** Representative FACS plots of Foxp3 expression in OT-II cells. **(B)** Bar diagram with frequencies of Foxp3+ CD4 cells from **(A)**. **(C)** Foxp3+ frequencies among the adoptively transferred CD4+ OT-II cells after PI-88 administration. **(D,E)** FACS plots and bar diagrams for the frequencies of Foxp3+ CD4+ cells across different culture conditions. **(F)** Percentage of Foxp3+ cells from the Treg induction experiment in **(D)**. **(G,H)** Treg percentages following Treg induction across indicated PG545 concentrations of **(I,J)**. Treg differentiation assay with decreasing concentrations of TGFβ. Experiments A–I are representative experiments that were each performed at least three times. **(K,L)** Treg differentiation assay of naïve human CD4+ cells in the presence of PG545 for 96 h. Representative of three independent experiments with four donors in total. Data represent mean fold change ± SD for the condition with 50 ng/ml of TGFbeta; *p < 0.05, **p < 0.01, ***p < 0.001 vs. respective control by unpaired *t*-test or one-way ANOVA with multiple comparisons where appropriate.

Because CD44 expression is reported to influence the number of Foxp3+ Treg by promoting Treg homeostasis but inhibiting expansion (36, 37) we considered whether PG545 might impact Treg. We did indeed observe that PG545 induced more Foxp3+ cells among OT-II T cells recovered from the spleen and draining lymph nodes of recipient mice. This was seen for a representative staining example (Figure 2A) as well as across samples from multiple mice (Figure 2B). We believe that these cells represent *de novo* induced Foxp3+ cells rather than the expanded pre-existing Tregs, since the transferred naïve OT-II cells have undergone multiple rounds of division before expressing Foxp3 (Supplemental Figure 1F). In comparison to these effects with PG545, we did not observe an increase in Treg upon treatment with either another heparan sulfate mimetic PI88 (Figure 2C) or with heparan sulfate itself using the same OT-II transfer model (data not shown). These data suggest that PG545 promotes the induction of Tregs *in vivo*. Given recent reports of the anti-inflammatory properties of PG545 in several systems (9, 22), we asked whether this agent promotes expansion of FoxP3+ Treg *in vitro*. To interrogate the effect of PG545 on T cells, we performed a Treg induction assay using a previously established protocol (33, 38). Addition of PG545 increased the percentage of Foxp3+ Treg in the presence of IL-2 and TGFβ (Figures 2D,E). In addition, the total frequencies of Foxp3+ cell per sample were also increased in the presence of PG545 (Figure 2F). PG545 promoted Treg induction at a range of concentrations (Figures 2G,H). PG545 was more effective at promoting Foxp3+ Treg induction at lower TGFβ concentrations (Figures 2I,J) indicating a high sensitivity of T cells to PG545 during suboptimal Treg differentiation conditions.

To test whether PG545-induced Foxp3+ cells were functional, we used a previously described *in vitro* suppression assay that uses induced Foxp3+ Treg in conjunction with autologous, CFSE-labeled CD4+ T cells and autologous irradiated CD4- cells as antigen presenting cells (APC) (39). Using this system, we determined that Tregs induced in the presence of PG545 were equally potent in suppressing effector T cell activation *in vitro* (Supplemental Figure 1E).

We also tested the effects of PG545 on human cells. PG545 favored Treg induction of naïve human CD4 cells, most potently at low concentrations of TGFβ (Figures 2K,L).

Together, these data demonstrate that PG545 promotes the expansion of both human and mouse Treg numbers *in vitro* and *in vivo*. Moreover, these data suggest that iTregs derived in the context of PG545 are functionally competent.

### PG545-Mediated Effects on Treg Induction Do Not Depend on HPSE Expression

We next asked whether the effect of PG545 on Treg induction was dependent on HPSE expression. To this end, we repeated our *in vitro* Treg induction assay using CD4+ T cells isolated from heparanase knock-out mice (HPSE^−/−^) mice. To interrogate this, we bred HPSE^−/−^ mice on a C57Bl6 background to strain-matched mice expressing a GFP/Foxp3 reporter (26, 27).

**Figure 3:**
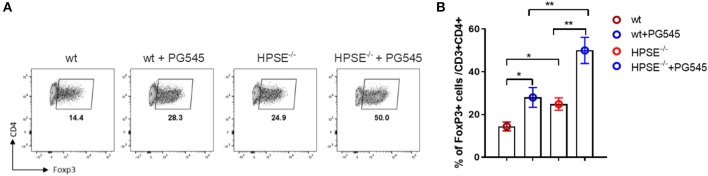
PG545 acts on Treg in heparanase-independent manner. **(A)** Treg differentiation of wild type and HPSE^−/−^ CD4 T cells in the presence of PG545. **(B)** Bar diagrams showing the frequencies of Foxp3+ CD4+ cells. Data are representative of three independent experiments. Mean ± SD, two-tailed unpaired *t*-test. *p < 0.05, **p < 0.01.

We found that PG545 doubled Foxp3 frequencies of wild type as well as HPSE-deficient CD4 cells (Figures 3A,B). Interestingly, HPSE^−/−^ CD4 T cells on their own produced higher frequencies of Foxp3+ cells, indicating that HPSE protein could inhibit iTreg induction. Nonetheless, the Treg-promoting effect of PG545 seems to be heparanase-dependent.

### PG545 Inhibits Erk Signaling in Lymphoid Cells

We then asked how PG545 might promote Treg induction but inhibit Th1 and Th17 polarization. Erk1/2 signaling is known to inhibit Foxp3+ Treg induction and to promote Th1 polarization via suppression of Foxp3 and RORγt expression (40–42). We therefore asked whether PG545 might impact Erk1/2 signaling. To address this question, we examined Erk1/2 phosphorylation in CTLL2 T cells.

**Figure 4:**
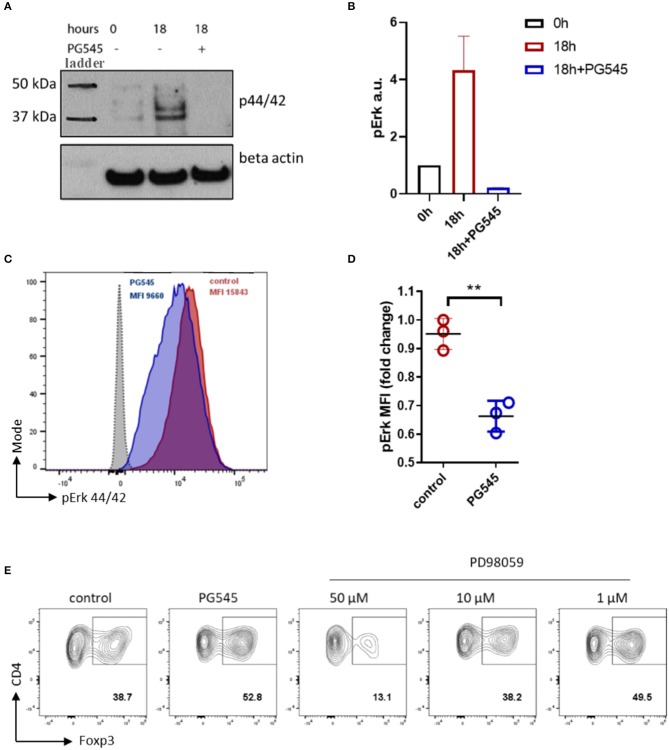
PG545 inhibits Erk signaling in lymphoid cell line and primary CD4 cells. **(A)** Immunoblot of pErk1/2 in 6 h serum starved CTLL2 cells. Time = 0 h indicate pErk in serum starved cells before adding the medium. 18 h = 18 h of incubation in CTLL2 medium ±PG545. **(B)** Densitometry of pErk signal against beta actin. **(C)** Phospho-flow analysis for pErk44/42 of primary CD3/CD28 activated mouse CD4 cells in the presence of PG545 for 72 h. **(D)** MFI quantification of pErk1/2 expression. Data are representative of experiments that were each performed at least three times. The fold change was normalized to 1. **(E)** iTreg differentiation in the presence of the selective inhibitor of MAPKK PD98059. Shown is the representative FACS plots, gate is on live CD3+ CD4+ lymphocytes. Data represent mean ± SD; *p < 0.05, **p < 0.01 vs. respective control by unpaired *t*-test.

We observed a potent inhibition of basal Erk1/2 phosphorylation after treatment with PG545 (Figures 4A,B). Similarly, PG545 strongly diminished pErk signaling in primary activated mouse CD4 T cells (Figures 4C,D). These data suggest that PG545 inhibits basal Erk1/2 signaling. In agreement with previous findings (40, 41), we observed a significant increase of Foxp3+ frequencies in the presence of Erk inhibitor (Figure 4E; Supplemental Figure 1G). This effect was dose-dependent, as higher concentrations of inhibitor suppresses Foxp3 induction and caused higher cell death (not shown). These findings are highly consistent with our observations that PG545 promotes Treg expansion and prevents polarization toward Th1.

### PG545 Inhibits Th17 and Promotes Treg Cells *in vivo*

**Figure 5:**
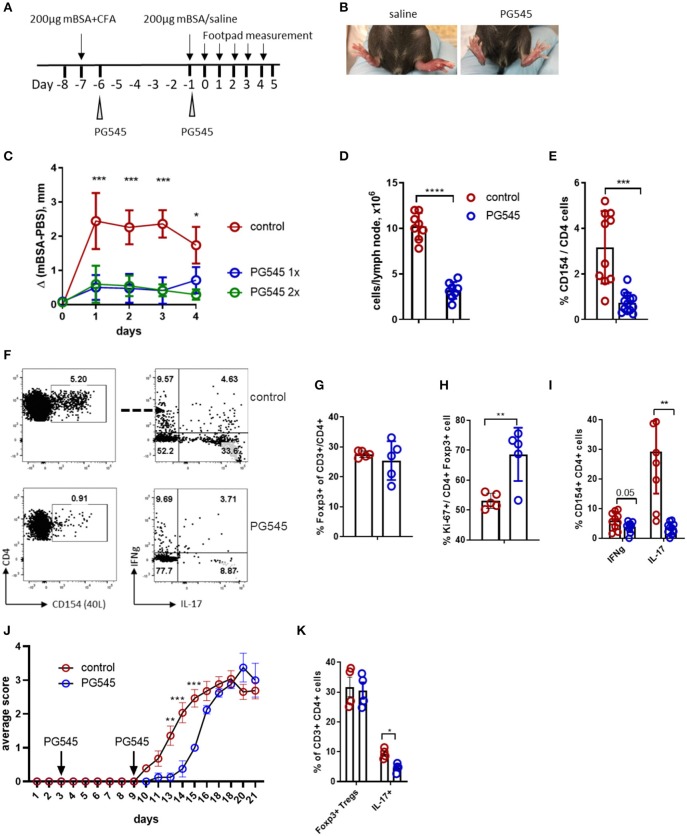
PG545 inhibits DTH response through decreasing Th17 potential. **(A)** Schematics of the DTH experiment. **(B)** Image of the footpad swelling in control and PG545-treated mice. **(C)** Footpad swelling shown as the difference between the control (saline) and mBSA footpad of each individual mouse. **(D)** Draining lymph node cellularity from mice in the DTH experiment. **(E)** Frequencies of the CD154+ cells after re-stimulation with mBSA in the presence of Brefeldin A for 6 h. **(F)** Representative flow cytometry for the T cell analysis from the popliteal (draining) LN of DTH mice. **(G)** Frequencies of Tregs in the draining LN. **(H)** *Ex vivo* proliferation of Tregs in the draining lymph node of mice. **(I)** Cytokine profile of CD154+ (antigen-specific) CD4 T cells from DTH experiments. **(J)** Clinical score of EAE in mice treated with PG545 and control mice. *n* = 11 for control, *n* = 5 for PG545. Statistical significance was assessed using mixed-effect analysis with multiple comparisons. **(K)** Frequencies of Tregs and Th17 cells in the spinal cord infiltrates of EAE animals. Bar diagram represent two independent experiments. Experiments A–I are representative experiments that were each performed at least three times. Data shown are for mean ± SD using a two-tailed unpaired *t*-test. *p < 0.05, **p < 0.01, ***p < 0.001, ****p < 0.0001.

We next sought to evaluate the functional impact of PG545 in a model of Th1/Th17-dependent inflammation. One such model is the methylated bovine serum albumin (mBSA) induced delayed type hypersensitivity (DTH) model (34). Here, the sensitization phase is initiated by immunization with mBSA, which is delivered subcutaneously as an emulsion with complete Freund's adjuvant (CFA). The elicitation phase of the reaction is then triggered by a second injection of mBSA into the hind paws. Footpad inflammation is then measured 24 h later. To test the impact of PG545 in this system, mice were treated with PG545 via a series of i.p. injections. A schematic of this protocol is shown in Figure 5A.

We observed that mice treated with PG545 showed a dramatically blunted hypersensitivity response (Figures 5B,C). Of note, there was no difference in footpad swelling between mice that received a single injection of PG545 1 day post-sensitization vs. those that received an additional injection on the day of challenge (elicitation phase) (Figure 5C).

To assess the effect of PG545 on priming vs. recall response, we treated DTH-immunized mice with PG545 at different time points. For this analysis, we also included HPSE^−/−^ mice, previously used in the iTreg induction experiment (Figure 3A). Remarkably, irrespective of the treatment protocol, PG545 potently suppressed footpad swelling in all the experimental groups (Supplemental Figure 2A). While HPSE^−/−^ mice mounted a normal DTH response, a single PG545 administration at day−6 fully prevented the swelling. Together these data demonstrate that PG545 has potent therapeutic effects in this model, irrespective of the administration.

We also assessed the impact of PG545 on the immune profile of these animals in this model. PG545 treated mice had a decrease in the draining LN cellularity (Figure 5D). This was accompanied with the decreased numbers of CD154+ CD4+ antigen-specific memory cells in the PG545-treated mice (Figures 5E,F). PG545 did not affect the systemic frequencies of Foxp3+ Tregs in this model (Figure 5G), but increased the proliferation of Tregs (Figure 5H).

Finally, we assessed the impact of PG545 on T cell polarization in this model. The fraction of IL-17 producing CD154+ T cells was dramatically diminished (Figures 5F,I). This also correlated with the cytokine staining obtained with the memory cell from the draining lymph node (Figure 1E). Likewise, memory cells from the mice treated at different times points all had impaired IL-17 production (Supplemental Figure 2B).

Since PG545 exhibits a strong inhibition of Th17 lineage, we wondered if this was the case for another Th17-mediated autoimmune model experimental autoimmune encephalomyelitis (EAE). Surprisingly, PG545 administration was not able to make mice resistant to EAE, although it did significantly delay its onset (Figure 5J). Interestingly, analysis of the spinal cord infiltrate revealed decreased frequencies of Th17 cells in mice treated with PG545, whereas Tregs remained unchanged (Figure 5K; Supplemental Figure 2C). This supports our observation in DTH model with respect to Th17 inhibition, but not the overall immune suppression.

Together these data indicate that PG545 is a potent inhibitor of DTH responses in mice and that PG545 may act in part by suppressing memory Th17 differentiation and priming.

### PG545 Inhibits LPS Mediated Dendritic Cell Maturation

**Figure 6:**
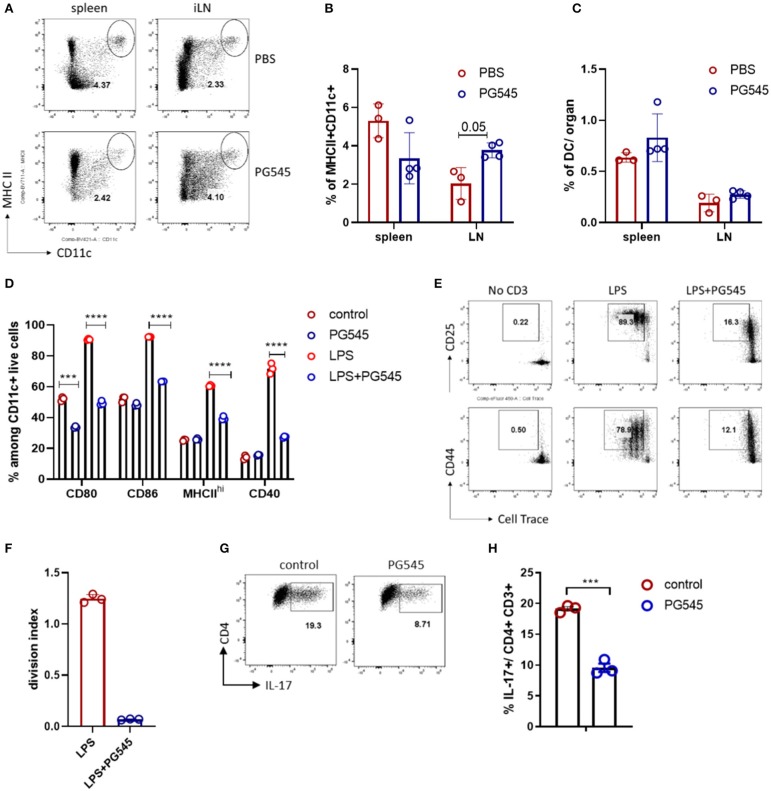
PG545 inhibits LPS response of dendritic cells (DC). **(A)** FACS plot depicting dendritic cell staining in PBS and PG545 treated mice. The gate is Zombie Aqua- CD3-B220^lo^ lymphocytes. **(B,C)** Bar diagrams with relative and total DC frequencies. **(D)** Bar diagram depicting maturation/activation markers on BMDC after LPS/PG545 stimulation for 24 h. Representative of three independent experiments. **(E,F)** Activation markers expression and proliferation of CD4 T cells after co-culture with BMDC from **(D)** for 3 days. Shown is one out of two experiments. **(G,H)** Th17 differentiation in the presence of BMDC. Data are expressed as mean ± SD. **(B–D)** were analyzed using two-way ANOVA with Turkeys's multiple comparisons; **(F,H)**- two-tailed unpaired *t*-test. *p < 0.05, **p < 0.01, ***p < 0.001, ****p < 0.0001.

Considering the very potent anti-inflammatory effect of PG545 irrespective of the administration regimen, we wondered if PG545 could have an effect on the antigen presenting cells. For this, we administered PG545 into naïve mice and assessed their dendritic cell compartment 7 days later. After *in vivo* PG545 administration to naïve mice we observed lower relative DC frequencies in the spleen, probably due to splenomegaly (Figures 6A,B), but slightly higher frequencies in the lymph node relative to B220^lo^ CD3- cells. In contrast, DC frequencies relative to total live cells in both the spleen and lymphatic tissue were unchanged (Figures 6A–C). Next, we sought to analyze the DC response to LPS in the presence of PG545. For this, we generated bone marrow-derived dendritic cells and assessed their phenotype and functionality *in vitro* in the context of PG545. Strikingly, while the treatment with PG545 alone did not have a major effect of BMDC phenotype, PG545 greatly diminished their LPS response as demonstrated by the decrease of maturation markers (Figure 6D). This is in contrast to the activation effect of PG545 on CpG treated BMDCs which was previously reported by Brennan et al. (23). Interestingly, we also observed a synergistic effect of PG545 and CpG toward DC maturation (data not shown).

In addition, while control BMDC potently activated T cells and promoted their proliferation in a polyclonal manner, PG545-treated BMDC failed to do so (Figures 6E,F). Since the inhibition of BMDC could contribute to the reduced polarization toward Th17 phenotypes observed in our *in vivo* models, we also assessed the Th17 polarization in the presence of BMDC. We find that the fraction of Th17 cells after *in vitro* activation was reduced in the setting of PG545 (Figures 6G,H).

### PI-88 and PG562 Favor Treg Induction *in vitro* but Do Not Suppress DTH

**Figure 7:**
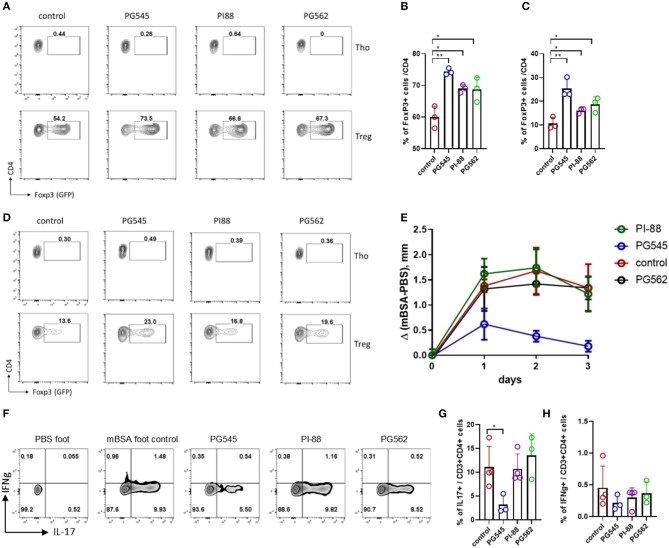
PI-88 and PG562 promote Treg induction but do not affect DTH response. **(A)** Treg induction assay in the presence of HS mimetics (all at 20 μg/ml) from mouse naïve CD4+ cells upon CD3/28 activation and TGFβ. **(B)** Aggregate bar diagram depicting Foxp3+ cell frequencies. **(C,D)** Aggregate bar diagram and FACS plot of Treg induction as in **(A)** from naïve CD4 cells co-cultured with mouse bone marrow derived dendritic cells (BMDC) in the presence of soluble aCD3 and TGFβ. **(E)** Footpad swelling depicted as the difference between the control (saline) and mBSA footpad of each mouse treated with HS mimetics (two injections, 400 μg/mouse). Mean ± SD, one-way ANOVA with Turkey *post-hoc* test. **(F)** Cytokine staining of the PMA/Ionomycin stimulated lymphocytes from the draining LN of DTH mice. Shown is the CD3+ CD4+ live dye+. Day 4 after mBSA challenge. **(G,H)** Summarized IL-17 and IFNg staining of the DTH mice. *p < 0.05, **p < 0.01, ***p < 0.001.

We also tested the effect of PI-88 and PG562 on Treg induction and T-cell mediated response. Both PI-88 and PG562 promoted Treg induction *in vitro* (Figures 7A–D) although less potently than PG545. This was true whether the Tregs were induced in the presence of anti-CD3/CD28 with TGFb (Figures 7A,B) or BMDCs with soluble anti-CD3 and TGFb (Figures 7C,D).

However, only PG545 was able to prevent footpad inflammation in a DTH model when all three components were tested side by side (Figure 7E). In agreement with the previous experiments, PG545 administration effectively suppressed the recall Th17 response in the draining LN of the animals, but neither PI-88 nor PG562 affected Th17 cells (Figures 7F,G). Of note, IFNg+ cells were not changed (Figure 7H).

Together these data indicate that while all three heparinase inhibitors tested promote Treg induction *in vitro*, PG545 is unique in its ability to inhibit Th17-mediate DTH responses in mice.

## Discussion

Here we report that the HS mimetic PG545 affects T cell differentiation and function. In particular, PG545 selectively promotes the induction of anti-inflammatory Tregs while inhibiting the development of Th17 both *in vitro* and *in vivo*. Consistent with these effects, PG545 inhibits Erk1/2 signaling, a pathway known to inhibit Foxp3+ Treg induction and promote Th1 and Th17 polarization (40–42).

These data expand on our understanding of PG545 beyond its impact on tumor models, where it decreased angiogenesis and metastasis and improved survival (43–46). More recently, several groups have shown a beneficial effect of PG545 treatment in atherosclerosis (47) and acute kidney injury (22). These effects were attributed to the reduction of pro-inflammatory markers, though neither increased Foxp3+ Treg nor reduced Th17 numbers were implicated.

Moreover, it appears that PG545 has both heparinase dependent and independent effects. Previously, the anti-angiogeneic effects of PG545, for example, were attributed to inhibition of heparinase and heparan sulfate-binding angiogenic growth factors (17). However, our data indicate that PG545 promotes the expansion of Foxp3+ Treg independently of heparanase. Consistent with this, PG545 was recently reported to have apoptotic effects on lymphoma cell lines that lack heparanase expression (2).

One question that this study raises is how an anti-inflammatory effect of PG545 observed here reconciles with its anti-tumor activity shown in other studies. Since cytotoxic T cells are the main effector T cells involved in cancer immunotherapy, it may be that these are less sensitive to the effect of PG545 treatment than tumor cells (48). It may also be that the effects of PG545 are context dependent. A recent study has shown that MEK inhibition can reactivate tumor-infiltrating CD8 lymphocytes by preventing their exhaustion (48). Finally, it may also be that the impact of PG545 on Erk signaling or other pathways in those models may trump effects on lymphocytes.

It is tempting to consider that both the anti-tumor and anti-inflammatory properties of PG545 may be attributable in part to inhibition of Erk1/2 signaling. However, PG545 has effects that are difficult to entirely attribute to Erk1/2 inhibition. For example, PG545 is reported to decrease glucose uptake, downregulate glycolytic machinery, inhibit Wnt signaling, and induces autophagy in cancer cell lines (2, 43, 44). Moreover, while Erk1/2 signaling inhibits Th17 polarization in some settings, it promotes Th17 in others, suggesting that both the regulation of these signaling cascades and their inhibition is likely to be complex (40, 49–51). Finally, we demonstrate effects similar to Erk signaling on Foxp3+ Treg but do not conclusively demonstrate that these are Erk-mediated. In the aggregate, our interpretation of the data is that it suggests that PG545 may acts via multiple mechanisms of which Erk1/2 inhibition is a part.

A report from Brennan et al. (23) suggested an activation effect of PG545 on dendritic cells. However, while Brennan et al. activated BMDC with the CpG mimic we used LPS stimulation, indicating the context-dependent properties of the HS mimetic.

Overall, we show that PG545 promotes regulatory T cells *in vitro*. This effect is accompanied with the inhibition of Th17 cells and to some extent Th1 cells. PG545 acts on T cells in heparanase-independent manner and impairs Erk signaling in proliferating cells without affecting an early activation program. PG545 administration effectively suppressed DTH in mice. These findings may inform the adaptation of PG545 for clinical applications including in inflammatory pathologies associated with DTH.

## Data Availability Statement

All datasets generated for this study are included in the article/Supplementary Material.

## Ethics Statement

All experiments were approved by the Stanford IACUC.

## Author Contributions

IK and HK designed and performed the experiments. CM edited the manuscript and performed some experiments with SZ and JV. DL helped with BMDC experiments. IK and PB conceptualized the study and wrote the manuscript.

### Conflict of Interest

The authors declare that the research was conducted in the absence of any commercial or financial relationships that could be construed as a potential conflict of interest.

## Supplementary Material

Supplemental Figure 1 **(A)** Histograms of T-bet and RORgt expression in CD4 T cells at Th1 and Th17 condition, respectively. **(B)** Spleen enlargement in mice after PG545 treatment. **(C)** Mouse body weight after PG545 treatment. **(D)** Spleen cellularity in mice on PG545. **(E)** Treg suppression assay with mouse iTregs induced in the presence of PG545. Representative CFSE dilution of effector CD4 T cells after co-culture with control iTregs, PG545 iTregs or alone. **(F)** Foxp3+ Tregs frequencies among the transferred OT-II cells followed by immunization with OVA/alum. **(G)** iTreg induction in the presence of MAPK kinase inhibitor. Data shown are for mean ± SD using a two-tailed unpaired *t*-test. ^**^*p* < 0.01, ^***^*p* < 0.001.

Supplemental Figure 2 **(A)** Footpad swelling of DTH mice treated with different regimen of PG545. Data are from one experiment with 4–5 mice per group. **(B)** FACS profile of the CD4 T cell cytokine staining from DTH mice with various PG545 treatment. **(C)** Representative bar diagram with Foxp3+ and IL-17+ cell frequencies among spinal cord CD3+CD4+ T cells in EAE mice. Data shown are for mean ± SD using a two-tailed unpaired *t*-test or two-way ANOVA test. ^*^*p* < 0.05, ^**^*p* < 0.01.
