# Supplementary figures and images for: The Heparan Sulfate Mimetic PG545 Modulates T Cell Responses and Prevents Delayed-Type Hypersensitivity
Source: Front Immunol. 2020 Feb 6;11:132. doi: 10.3389/fimmu.2020.00132 (PMC7015948; doi:10.3389/fimmu.2020.00132)

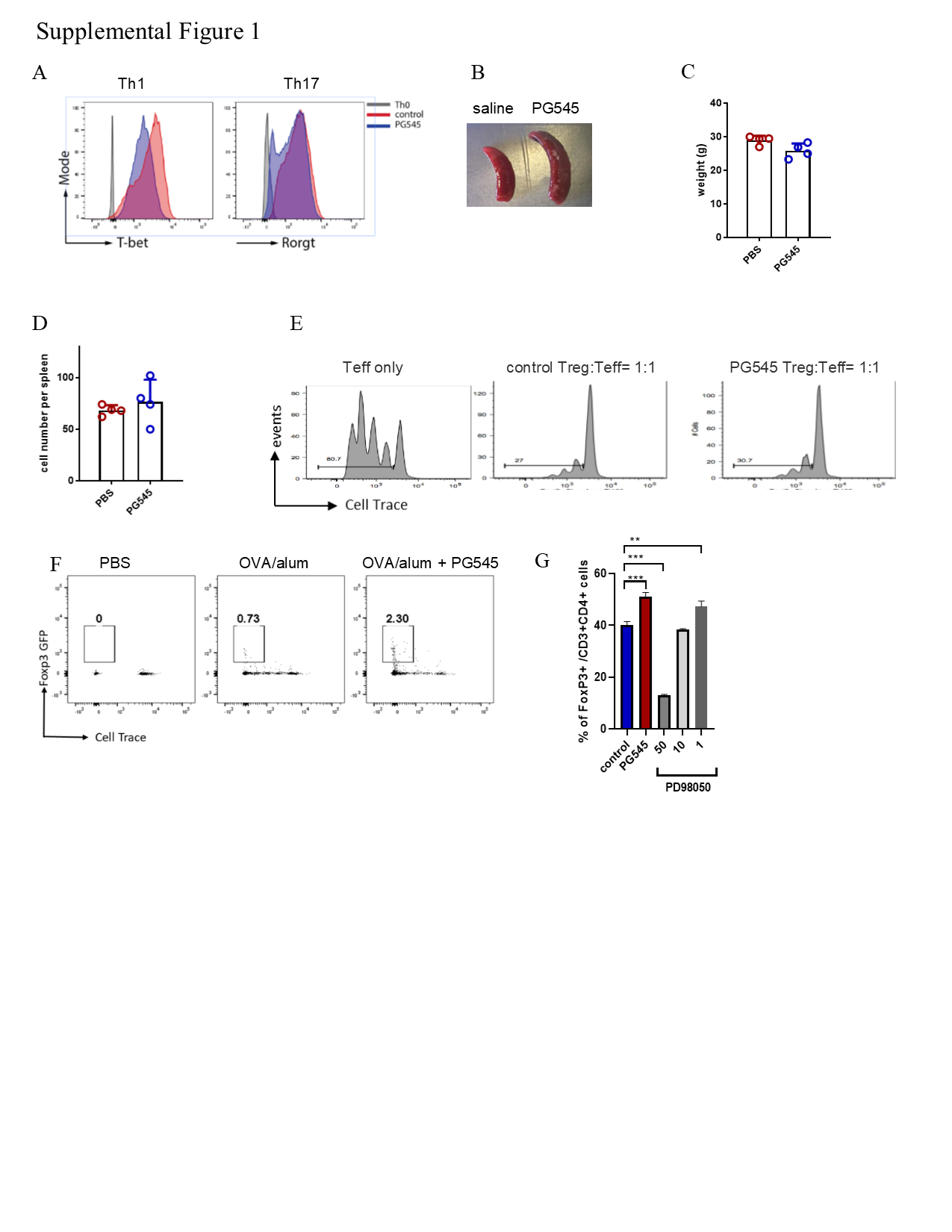

Supplement: Supplemental Figure 1 — (A) Histograms of T-bet and RORgt expression in CD4 T cells at Th1 and Th17 condition, respectively. (B) Spleen enlargement in mice after PG545 treatment. (C) Mouse body weight after PG545 treatment. (D) Spleen cellularity in mice on PG545. (E) Treg suppression assay with mouse iTregs induced in the presence of PG545. Representative CFSE dilution of effector CD4 T cells after co-culture with control iTregs, PG545 iTregs or alone. (F) Foxp3+ Tregs frequencies among the transferred OT-II cells followed by immunization with OVA/alum. (G) iTreg induction in the presence of MAPK kinase inhibitor. Data shown are for mean ± SD using a two-tailed unpaired t-test. **p < 0.01, ***p < 0.001. [file Image_1.tif]

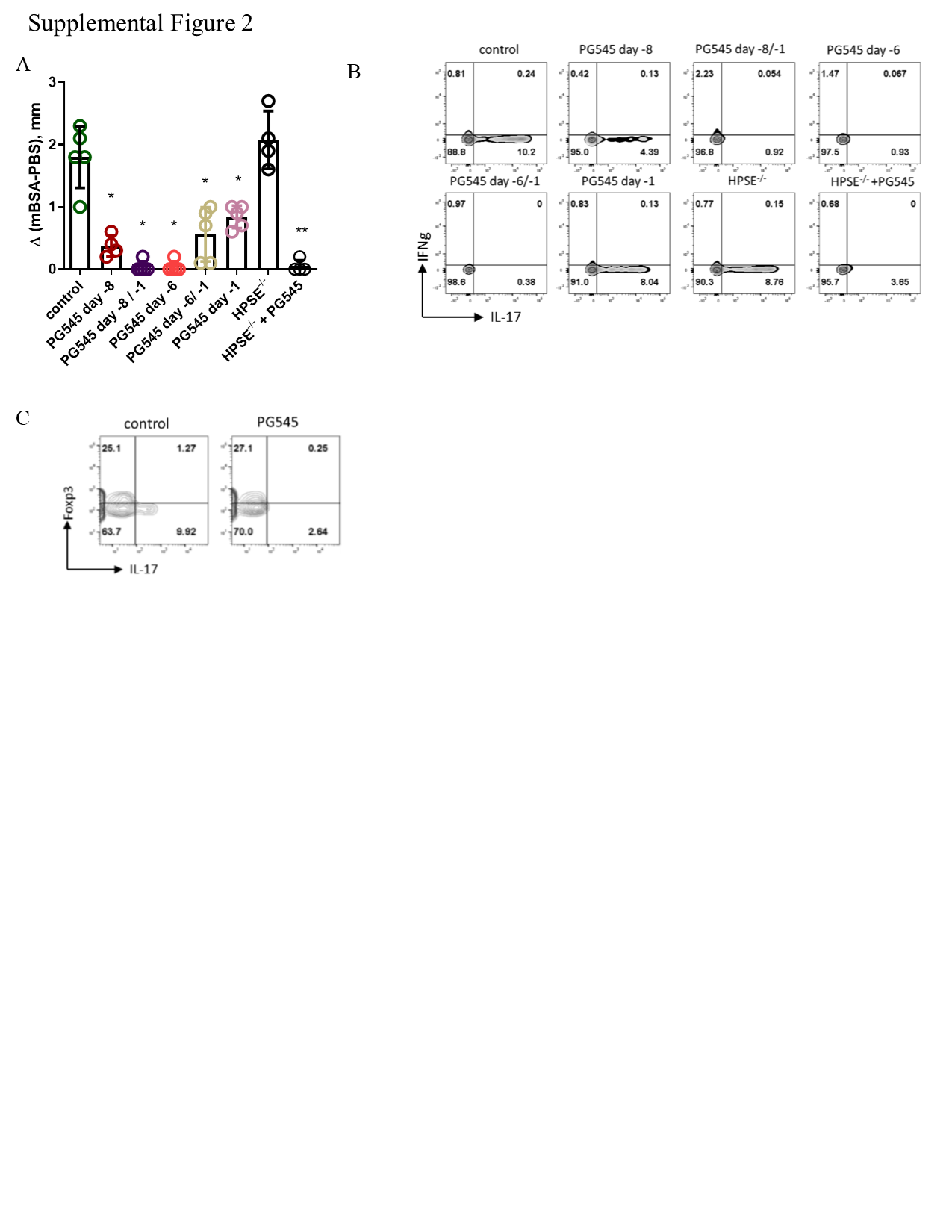

Supplement: Supplemental Figure 2 — (A) Footpad swelling of DTH mice treated with different regimen of PG545. Data are from one experiment with 4–5 mice per group. (B) FACS profile of the CD4 T cell cytokine staining from DTH mice with various PG545 treatment. (C) Representative bar diagram with Foxp3+ and IL-17+ cell frequencies among spinal cord CD3+CD4+ T cells in EAE mice. Data shown are for mean ± SD using a two-tailed unpaired t-test or two-way ANOVA test. *p < 0.05, **p < 0.01. [file Image_2.tif]
